# Study of oral microorganisms contributing to non‐carious cervical lesions via bacterial interaction and pH regulation

**DOI:** 10.1111/jcmm.16370

**Published:** 2021-02-16

**Authors:** Xiaoyu Huang, Lin She, Huanhuan Liu, Pingping Liu, Jue Chen, Yingcong Chen, Wenjie Zhou, Youguang Lu, Jun Lin

**Affiliations:** ^1^ Department of Preventive Dentistry School and Hospital of Stomatology Fujian Medical University Fuzhou China; ^2^ Fujian Key Laboratory of Oral Diseases & Fujian Provincial Engineering Research Center of Oral Biomaterial & Stomatological Key lab of Fujian College and University School and Hospital of Stomatology Fujian Medical University Fuzhou China; ^3^ Institute of Applied Genomics Fuzhou University Fuzhou China; ^4^ College of Biological Science and Engineering Fuzhou University Fuzhou China; ^5^ Fujian Key Laboratory of Marine Enzyme Engineering Fuzhou University Fuzhou China

**Keywords:** 16S rRNA, dental morphology, microbial interactions, non‐carious cervical lesions, oral microbiology

## Abstract

There is a lack of evidence about the relationship between microorganisms and non‐carious cervical lesions (NCCLs) due to limited technologies. A group of 78 patients was enrolled for microbial 16S rRNA sequencing of dental plaques on normal and defective cervical surfaces. Parallel data from 39 patients were analysed with paired *t* tests, and *Fusobacteriales* exhibited significantly less distribution on NCCLs than on normal surfaces. As a result, *Fusobacterium nucleatum*, the most common oral bacterial strain belonging to the order *Fusobacteriales*, was selected for further research. From a scanning electron microscopy (SEM) scan, the tooth surface with *Fusobacterium nucleatum* and *Streptococcus mutans* culture was more intact than that without *Fusobacterium nucleatum*. Furthermore, the calcium contents in groups with *Fusobacterium nucleatum* were significantly higher than that without it. In further mechanistic research, *Fusobacterium nucleatum* was demonstrated to adhere to and disturb other organisms as well as producing alkaline secretions to neutralize the deleterious acidic environment, protecting the tooth structure. In conclusion, microorganisms and NCCLs were confirmed directly related through adherent bacterial interactions and pH regulation. The research provides a new perspective and experimental evidence for the relation between microorganisms and NCCLs, which guides clinical treatment and preventive dentistry in the future.

## 1 INTRODUCTION

A NCCL is defined as loss of dental hard tissue at the cementoenamel junction (CEJ), which is commonly encountered in clinical practice.^1^, ^2^ The prevalence of NCCLs has not been well documented because of varying investigation methods and samples; thus, it is difficult to compare the results obtained from diverse scientific studies.^3^, ^4^ According to a study in 1985, non‐carious destruction contributed to nearly 25% of the pathological processes of hard dental tissue.^5^ However, it is well recognized that their prevalence and severity increase with age, and the first premolars are affected most frequently.^6^, ^7^

Dental lesions at the neck produce a series of unpleasant symptoms, such as sensitivity, pain and unaesthetic appearance. The problems draw increasing attention as the life expectancy of humankind increases and awareness of the importance of oral health increases.^8^ The most commonly acknowledged aetiology causing the progression of NCCLs on the neck is multifactorial, including erosion, abrasion and occlusal trauma.^3^, ^4^ Evidences suggest that abrasion and erosion from long‐term physical‐chemical effects could lead to the destruction of the dental crystalline structure of hydroxyapatite and fluorapatite. Lee et al proposed the occlusal stress and tooth flexion theory, noting that traumatic occlusion results in tooth tissue deformation to create microcracks under the magnitude and direction of masticatory forces.^9^

A comprehensive understanding of the impact of the oral microbiota on host health and disease is considered a holistic view of intra‐ and cross‐kingdom interactions among members of the oral microbiome, and imbalance of the oral flora leads to caries, periodontal disease and other oral pathologies.^10^, ^11^ Due to the special oral environment of the cervical teeth, anatomical structure changes the wedge‐shaped defects, residual food and unqualified cleaning can bring about corresponding changes in the local microecology. In recent years, most surveys involving wedge‐shaped defects have focused mainly on the occlusion and comparison of different filling materials and methods.

In fact, there is a lack of definite evidence to demonstrate that microorganisms do not lead to NCCLs or that no microflora variation occurs in NCCLs. Considering that the official definition of NCCLs denies the function of caries, few researchers are interested in the relationship between oral microorganisms and defect sites.^2^, ^12^ Moreover, previous microflora studies were limited mainly by the inefficiency of technologies because of the extremely minimal flora existing on the smooth surface of tooth defects for most people without caries. The microbial community on the surface of teeth is diverse, so we are interested in conducting a systematic study on the correlation between defects and microorganisms. The rapid development of high‐throughput sequencing technologies in the past decade provides scientists with new perspectives as well as opportunities to explore the composition of the microbial world and the unknown area of life science.^13^, ^14^

In this study, microorganism specimens from the facial surface of the cervical tooth in subjects with healthy tooth surfaces and those with wedge‐shaped defects were collected, measured and analysed. Furthermore, some functional experiments were conducted in vitro to investigate the correlation of dental wedge‐shaped defects and oral microbes, especially whether oral microbes could affect the occurrence and development of wedge‐shaped defects. The aim of this study was to explore the role of oral microbes in NCCLs and provide an experimental basis for NCCLs prevention and treatment.

## 2 MATERIALS AND METHODS

### 2.1 Clinical samples

Seventy‐eight patients with a diagnosis of wedge‐shaped defects were selected from the Preventive Department of the Hospital of Stomatology, Fujian Medical University. Sterile periodontal scalers were used to scrape the dental plaque on the cervical surfaces of healthy and defective teeth. This procedure was immediately followed by immersion in 50 μL of Onestep direct DNA extraction reagent (Weiyin, China), which is able to treat tiny environmental samples containing microorganisms, and the samples were heated to 85°C for 15 minutes to prepare DNA for subsequent PCR.

The clinical protocol and inform consent were approved by the Ethics Committee of Institutional Review Board of School and Hospital of Stomatology, Fujian Medical University. The written informed consent was obtained from all subjects before interviews. Face‐to‐face interviews were performed by trained interviewers with a structured questionnaire, and all data were self‐reported by the interviewees.

### 2.2 PCR, next‐generation sequencing and 16S rRNA gene analysis

DNA extraction of specimens was accomplished by using Onestep direct DNA extraction reagent, followed by using bacterial 16S rRNA V3‐V4‐specific primers (upstream primer: 5′ CCTAYGGGRBGCAAG 3′, downstream primer: 5′ GGACTACNNGGGTATCTAAT 3′) to amplify the DNA template. PCR amplification was conducted in a total volume of 10 μL containing 5 μL of 2X Taq PCR Mix (CWbio, China), 0.4 μL of each primer (10 μmol/L), 0.5 μL of DNA template and 3.7 μL of ddH_2_O. PCR was performed under the following conditions: initial denaturation at 94°C for 3 minutes; 30 cycles of 94°C for 30 seconds, 53°C for 30 seconds, and 72°C for 30 seconds; and a final extension at 72°C for 3 minutes.

A Universal DNA Library Prep for ILLUMINA®V2 Kit (Vazyme, China) was used to prepare libraries of the PCR amplification products for next‐generation sequencing.^15^ The paired‐end 250 bp (PE250) mode of an Illumina HiSeq 2500 sequencer was implemented. Then, the sequencing results were analysed using the QIIME2 software package.^16^

### 2.3 Tooth collection

A sample of 10 freshly extracted human premolars was randomly selected from the Oral and Maxillofacial Surgery Department of Hospital of Stomatology, Fujian Medical University. The exclusion criteria of the chosen teeth included advanced caries lesions (ie International Caries Detection and Assessment System [ICDAS] index 6),^17^ abrasion and fracture.^7^

Upon receipt, these intact teeth first underwent professional periodontal curettage to remove dental calculus, pigmentation and attached soft tissue remnants. Subsequently, the teeth were kept in a 0.5% sodium hypochlorite solution for surface disinfection overnight and cleaned in a phosphate‐buffered saline (PBS) solution (pH 7.4).

### 2.4 Microbial culture

*Streptococcus mutan*s (ATCC25175) was cultivated using brain heart infusion (BHI) broth (Oxoid, UK). *Fusobacterium nucleatum* (ATCC10953) was inoculated in trypticase soy agar/broth with defibrinated sheep blood.

*Fusobacterium nucleatum* is an obligate anaerobe, while *Streptococcus mutans* is a facultative anaerobe. Both microorganisms used in this study were cultured under anaerobic conditions with a biobag (Thermo AnaeroGen, USA) at 37°C.

### 2.5 Specimen treatment

Either the tooth crown or root was transversely and partly cut off using a rotating highly concentrated diamond saw (Buehler, USA) under water cooling. The retained tooth tissue portion was also sectioned in half parallel to the long axis of the teeth, from buccal to lingual surfaces, by the cutting wheel. Finally, the tooth slices were stored in sterile artificial saliva (Chuangfeng Technology, China) at 4℃. The two equivalent tooth slices obtained from each tooth were sorted into two groups with differently appointed numbers. For example, half of the tooth specimen was named 1A, 2A…10A in the control group, while the other half was named 1B, 2B…10B in the treatment group.

The specimens of 1B, 2B…5B were placed in five conical flasks containing only *Streptococcus mutan*s suspensions, while the specimens 6B, 7B…10B were placed in another five conical flasks containing *Streptococcus mutan*s following *Fusobacterium nucleatum* culture, where they remained separately for 6 days at 37℃ under gentle agitation. Afterwards, the abovementioned dentinal cylinders were removed from the flasks and cleaned with PBS.

### 2.6 pH value variation

After incubation, the pH values of *Fusobacterium nucleatum* and *Streptococcus mutans* cultures were detected by a pH meter after 2, 4 and 6 days (Mettler, Switzerland). The culture conditions were the same as those above.

### 2.7 SEM and outcome measure

After inoculation for up to 6 days, the treated specimens were prepared for SEM examination. Dentinal cylinders were fixed in 35% glutaraldehyde and dried with ascending ethanol concentrations.^18^ Afterward, they were dehydrated to their critical point with a drying apparatus (Bio‐Rad, E3000, Watford, UK), fixed on microscope slides and gold‐coated using a sputter coater (Bio‐Rad). The surface morphology of the tooth specimens was captured by a tungsten filament SEM (Quanta 250*, FEI, USA). EDX spectroscopy mapping was performed by using an XFlash 6130 microscope (Bruker, Germany) to identify the chemical compositions of the surface of crystal phases.

### 2.8 Bioinformatics and statistical analysis

Raw reads were demultiplexed and quality filtered by QIIME 2, a next‐generation microbiome bioinformatics platform, to determine the alpha and beta diversity indexes.^16^ The alpha and beta diversity data were performed using Wilcoxon rank‐sum test.

Quantitative data are shown as the mean ± standard deviation (SD), median or interquartile range, while qualitative data are presented as frequencies. Normally distributed continuous variables were processed through paired and 2 independent samples *t* tests.^19^ Univariate and multivariate linear regression models were applied to analyse the association between given factors and NCCL risk. A two‐tailed *P* < 0.05 was defined as statistically significant. All data analyses were performed with R software (version 3.6.0).

Models were built using forward variable selection. Variables explored included stable demographic factors and modifiable lifestyle factors.^20^

## 3 RESULTS

### 3.1 Sequencing analysis

**FIGURE 1:**
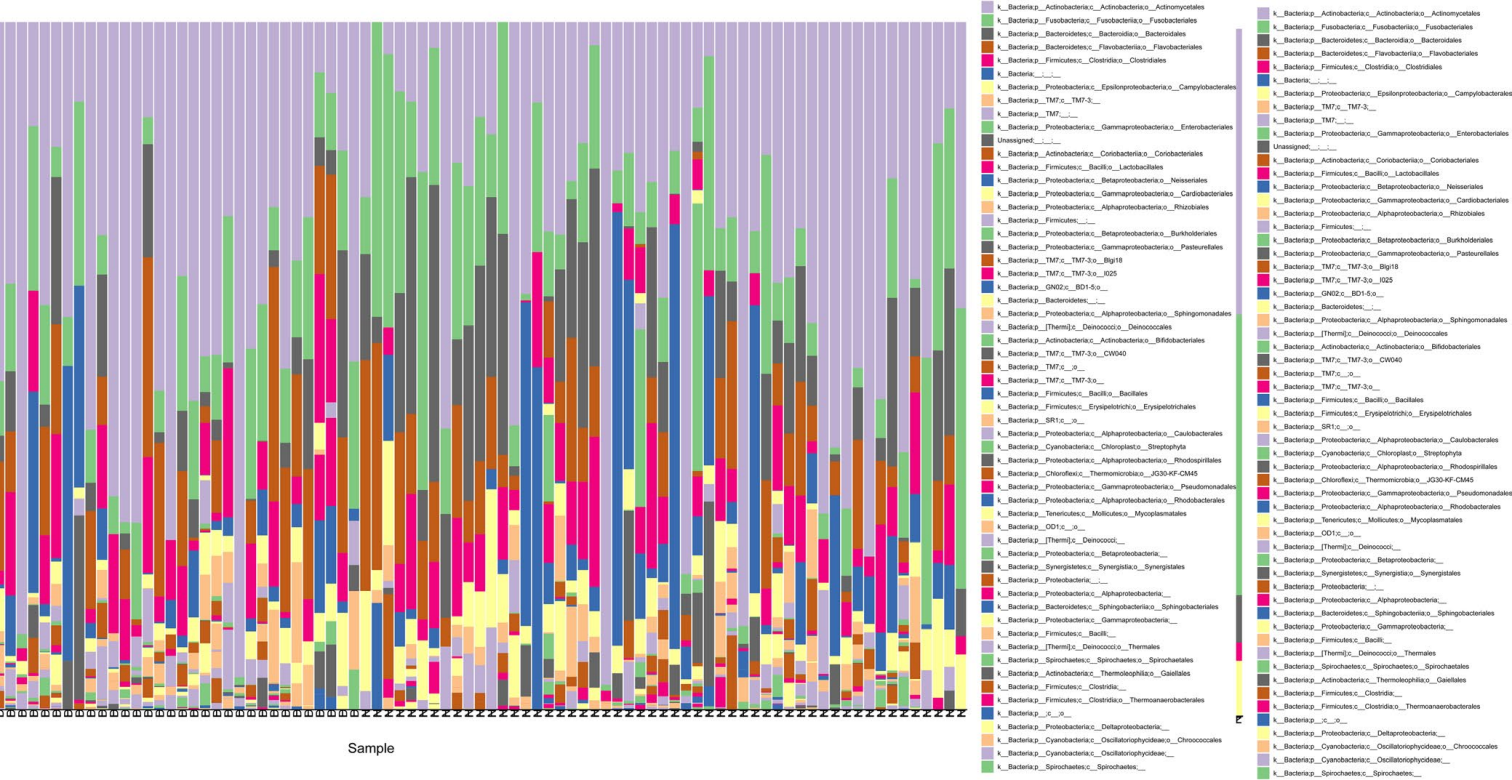
Descriptive overview of the dental microflora represented by a QIIME2‐generated bar plot of 16S rRNA‐identified bacterial taxa (n = 104). Across the 104 samples, the composition and diversity of microbial communities recruited on the tooth neck were characterized by a QIIME2‐generated bar plot

**FIGURE 2:**
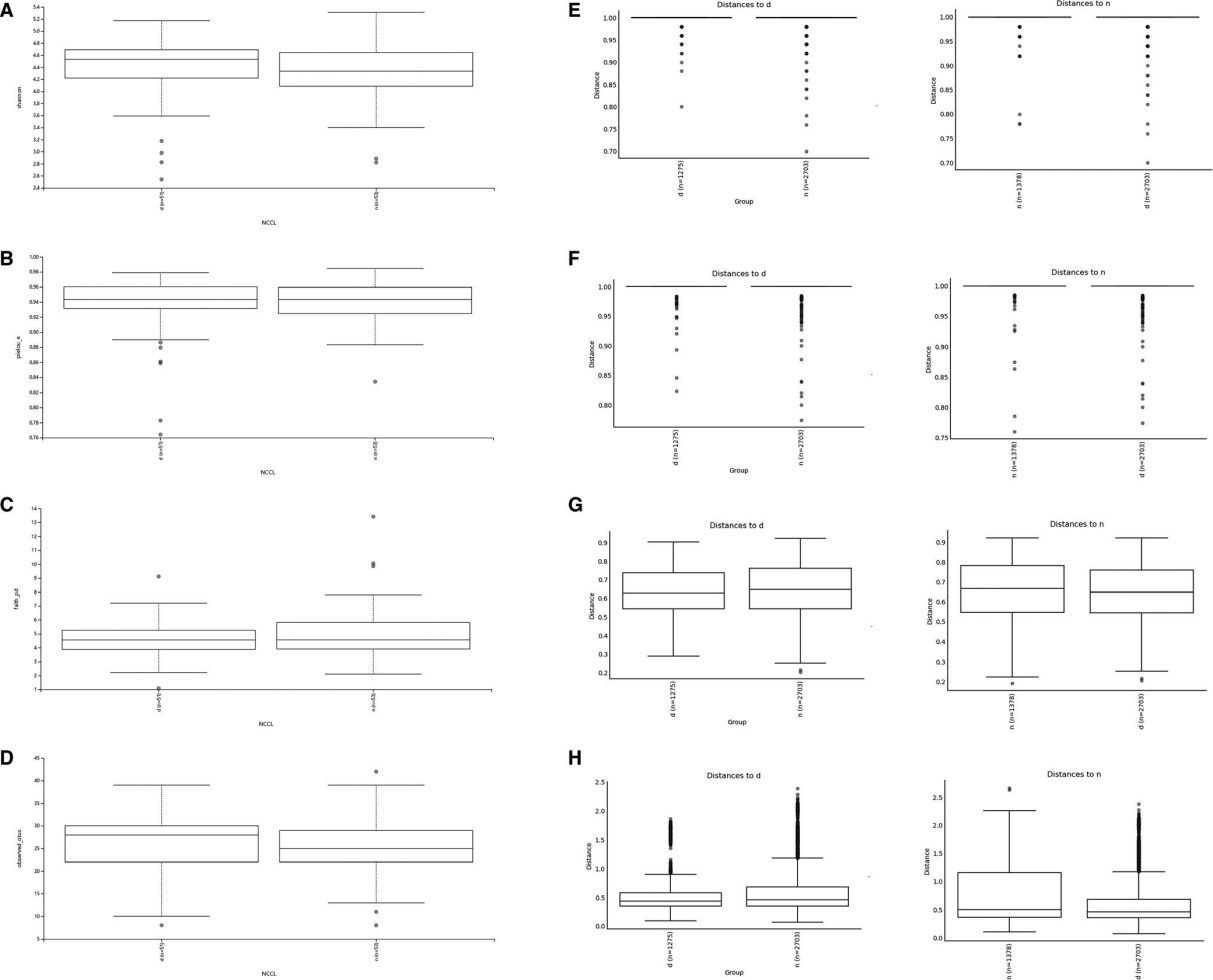
There were no significant structural differences in the alpha and beta diversity. (A‐D) Alpha diversity comparison in Shannon, Pielou_e, Faith_pd and Observed_otus index (n = 104, *P* >0.05). (E‐H) Beta diversity comparison in (E) Bray_curtis, (F) Jaccard, (G) unweighted without abundance and (H) unweighted with abundance index (n = 104, *P* >0.05). Statistical analysis of the data was performed using Wilcoxon rank‐sum test, and *P* > 0.05 represented for no significant differences

After DNA extraction and PCR, 104 dental plaque samples from 78 clinical patients were successfully amplified for 16S rRNA, while the other 52 samples were eliminated because of difficulty with PCR. The 16S rDNA V3‐V4 region amplicon sequencing generated 12 880 368 high‐quality Illumina PE250 reads, with an average of 123 850 reads per sample. The raw Illumina paired‐end read data for all samples have been deposited in the Short Read Archive under the accession number PRJNA589106. Across the 104 samples, the composition and diversity of microbial communities recruited on the tooth neck were characterized by a QIIME2‐generated bar plot (Figure 1). The richness, diversity and relative abundance of each taxon were determined with different indexes and algorithms.^21^ Regarding diversity, there were no significant structural differences in the alpha diversity indexes (Shannon, *P* = 0.180; Pielou_e, *P* = 0.997; Faith_Pd, *P* = 0.628; Observed_OTUs, *P* = 0.097, as shown in Figure 2A‐D), or beta diversity (Bray_curtis, *P* = 0.889; Jaccard distance, *P* = 0.859; unweighted UniFrac algorithms without abundance, *P* = 0.642; weighted UniFrac algorithms with abundance, *P* = 0.595, as shown in Figure 2E‐H), between the normal group and NCCL group, suggesting that their microbiota diversities were similar.^22^

Based on the Human Oral Microbiome Database (http://www.homd.org/index.php), 16 kinds of the most prevalent taxa at the phylum level were reported. Table 1 shows 10 of the 16 identified aggregated relative abundances at the phylum level (per cent total counts) in our study samples, which include *Actinobacteria* (27.1%), *Bacteroidetes* (22.5%), *Firmicutes* (11.2%), *Fusobacteria* (14.7%) and *Proteobacteria* (10.4%).

**TABLE 1:** Phylum‐level community composition for control, NCCL and total study samples. The abundance level was calculated for aggregated read counts across all samples for NCCLs classified at the phylum level (ie aggregates of all taxa with the same phylum assignment)

Phylum‐level taxonomy	Percent in control samples (%)	Percent in NCCL samples (%)	Percent across all samples (%)
*Actinobacteria*	26.6	27.5	27.1
*Bacteroidetes*	22.2	22.9	22.5
*Chloroflexi*	0.1	0	0.0
*Cyanobacteria*	0.0	0.1	0.0
*Firmicutes*	9.7	12.6	11.2
*Fusobacteria*	14.9	14.5	14.7
*Proteobacteria*	11.2	9.6	10.4
*SR1*	0.1	0.1	0.0
*Spirochaetes*	0.0	0.0	0.0
*Synergistetes*	0	0.0	0.0

### 3.2 Paired *t* test analysis

Eliminating unpaired cases of healthy and defective samples in the same patient, the available data were ultimately obtained from wedge‐defect samples and normal samples from 39 patients.

Through sequencing, the relative abundance of all strains in each sample was obtained. The relative proportion of each order was calculated and compared with a paired *t* test. As shown in Table 2, *Bacteroidales* and *Firmicutes* seemed to display an association with NCCL risk, although the association was not statistically significant (*Bacteroidales,* *P* = 0.056; *Firmicutes,* *P* = 0.066), while *Actinomycetales*, *Clostridiales* and *Fusobacteriales* exhibited statistically significant differences *(Actinomycetales,* *P* = 0.023; *Clostridiales,* *P* = 0.011). Among these, *Fusobacteriales* showed an extremely significant difference (*P* = 0.000345, *t* = 3.932) between the normal group and the NCCL group.^23^

**TABLE 2:** The *P* and *t* values of key oral bacterial taxa were calculated by paired *t* tests

Microorganism	*P* value	*t* value
*Actinomycetales*	0.023*	2.373
*Bacteroidales*	0.056	−1.975
*Clostridiales*	0.011*	2.667
*Fusobacteriales*	0.00035***	3.932
*Firmicutes*	0.066	1.89

*** *P* < 0.001,

** *P* < 0.01,

* *P* < 0.05.

### 3.3 Correlation analysis of NCCL and possible factors

In addition to the traditional aetiology, additional possible factors associated with oral organisms were speculated to explore the relationship among *Fusobacterium nucleatum*, lifestyle factors and clinical variables by univariate and multivariate linear regression. The related statistical analysis with coef., *P* values and 95% CIs of 39 survey respondents is described in Tables 3 and 4. As shown in Table 3, using univariate linear regression, there was no significant difference revealed between *Fusobacterium nucleatum* and most factors, such as sex, smoking history and alcohol consumption. Nevertheless, the occurrence of NCCLs was positively correlated with *Fusobacterium nucleatum* abundance (*P* = 0.013), which was consistent with our expectation. Similarly, a multivariate regression model in Table 4 further confirmed that NCCLs positively correlated with *Fusobacterium nucleatum* abundance (*P* = 0.040). In addition, the *P* value of tooth brushing frequency was close to 0.04, which indicated the traditional pathogenic factors related to tooth brushing.^20^

**TABLE 3:** Factors associated with *Fusobacterium* sp in a univariate linear regression model

Variable	Category	Coef.	*P*	95% CI
Sex	Male	Ref.		
	Female		0.518	−0.021‐0.042
Tooth brushing frequency (times/d)	1	Ref.		
	≥2		0.181	−0.047‐0.009
Smoking history	No	Ref.		
	Yes		0.312	−0.062‐0.020
Alcohol consumption	No	Ref.		
	Yes		0.697	−0.046‐0.031
Antibiotic use	No	Ref.		
	Yes		0.603	−0.043‐0.074
Capsaicin consumption	No	Ref.		
	Yes		0.129	−0.073‐0.009
Medical toothpaste use	No	Ref.		
	Yes		0.138	−0.011‐0.079
Soda consumption	No	Ref.		
	Yes		0.273	−0.108‐0.031
NCCL	No	Ref.		
	Yes		0.013*	0.008‐0.067

* *P* < 0.05.

**TABLE 4:** Factors associated with *Fusobacterium* sp in a multivariate linear regression model

Variable	Category	Reference	Coef.	*P*	95% CI
Tooth brushing frequency (times/d)	2	1	−0.031	0.040*	−0.060‐0.001
NCCL	Yes	No	0.034	0.040*	0.002‐0.068

* *P* < 0.05.

### 3.4 SEM and element mapping data of tooth surfaces after *Fusobacterium nucleatum* and *Streptococcus mutans* culturing

Relative to other destructive bacteria, *Fusobacterium nucleatum* was suspected to have protective effects on dental hard tissues. To simulate the oral microenvironment, we selected the most common damaging bacterial strain, *Streptococcus mutans,* and cultured it separately or followed with *Fusobacterium nucleatum* to explore and compare their effects.

**FIGURE 3:**
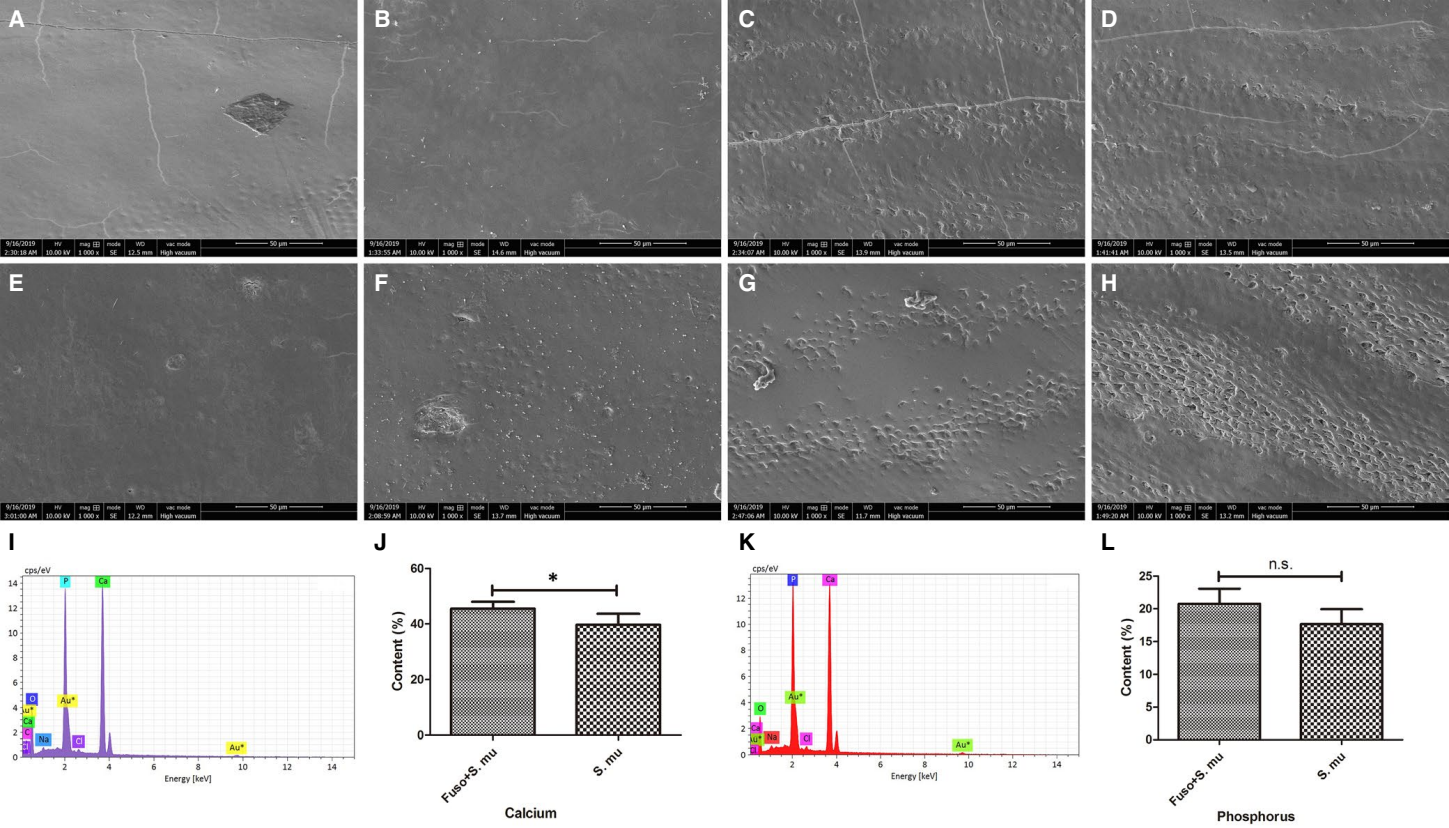
SEM images and element mapping results of tooth surfaces after *Fusobacterium nucleatum* and *Streptococcus mutans* culturing. A, C, E, G, Tooth slices without any treatment (n = 10). B, D, Tooth slices with *Fusobacterium nucleatum* and *Streptococcus mutans* coculture (n = 5). F, H, Tooth slices with only *Streptococcus mutans* (n = 5). I, The element mapping data of the *Fusobacterium nucleatum* coculture group. J, The comparison of calcium contents in the two groups. K, The element mapping data of the *Streptococcus mutans* alone. L, The comparison of phosphorus contents in the two groups. Error bars represent the standard deviation. Statistical analysis of the data was performed using Student's *t* test; n.s. represented for no significant difference (*P* > 0.05), while * represented for significant difference (*P* < 0.05)

Oral multispecies communities were formed on dentin specimens by culturing *Fusobacterium nucleatum* and *Streptococcus mutans*. At the end of the incubation period, images were obtained with a SEM (magnification: ×1000). In comparison with that of the tooth slices from the other half of the same tooth or those incubated with *Streptococcus mutans* culture alone, the surface morphology of the tooth specimens with only *Streptococcus mutans* exhibited the most distinct cracks (Figure 3A‐D). The most interesting appearance we detected from the specimens immersed in the *Fusobacterium nucleatum* and *Streptococcus mutans* culture revealed nearly similar morphology to that of untreated control slices (Figure 3E‐H). Since the specimens we selected were teeth isolated from oral cavities of different humans, the tooth surface itself was greatly affected by individual differences. Some teeth surfaces were relatively smooth, homogenous and shiny, while others originally suffered potential structural damage. Through random allocation, we selected both relatively smooth visual fields (Figure 3A,B,E,F) and relatively rough visual fields (Figure 3C,D,G,H) to objectively illustrate the possible influence of bacteria on the tooth surface. It was observed that *Fusobacterium nucleatum*, on both the relatively intact and relatively rough tooth surfaces, produced no significant changes in morphology compared with that of the control. In the *Streptococcus mutans* alone group, the relatively smooth tooth surface exhibited significant defects, while the originally rough surface showed widened and deepened destruction.

According to the elemental mapping data (Figure 3I,J), the calcium contents in groups with *Fusobacterium nucleatum* were significantly higher than those in groups with only *Streptococcus mutans* (*P* = 0.047, *t* = 2.491), which further supported that teeth were destroyed in the *Streptococcus mutans* group and protected in the *Fusobacterium nucleatum* group. Although the average phosphorus content in the only *Streptococcus mutans* groups was lower than that in the *Fusobacterium nucleatum* groups (Figure 3K,L), there was no statistically significant difference. As a result, it was concluded that *Streptococcus mutans* could act as an independent risk factor in tooth destruction. In contrast, *Fusobacterium nucleatum* might prevent structural integration and counteract the damage induced by *Streptococcus mutans*. Every group contained 5 specimens, and every test was performed 3 times.

### 3.5 *Fusobacterium nucleatum* adheres to other bacteria and affects the pH value

**FIGURE 4:**
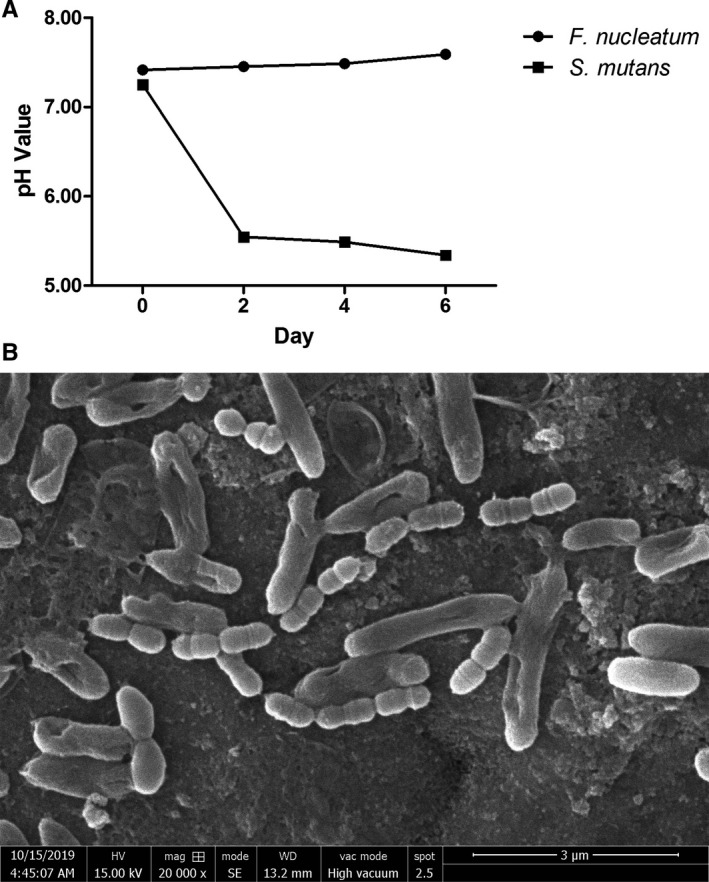
*Fusobacterium nucleatum* adheres to other bacteria and affects the pH value. A, The pH value variation in *Fusobacterium nucleatum* and *Streptococcus mutans* suspensions over 6 d (n = 3). Error bars represent the standard deviation. B, SEM of *Fusobacterium nucleatum* and *Streptococcus mutans* coculture (n = 3)

To further explore the mechanism of tooth tissue damage by *Streptococcus mutans* and protection by *Fusobacterium nucleatum*, SEM was utilized to visibly record the morphology and growth statuses of bacteria. As shown in Figure 4A, pure *Streptococcus mutans* was distinctively biojoint‐spherical shaped, while pure *Fusobacterium nucleatum* was long and rod shaped. When the two kinds of bacteria were cultured, we found that *Fusobacterium nucleatum* could adhere to *Streptococcus mutans,* which exhibited significant transformation and stretching at the contact region, as if a tentacle was extended to capture other bacteria.

Also, we conducted pH test experiments under different conditions. The BHI and sheep blood culture medium was slightly alkaline, and its pH values were approximately 7.2 to 7.4 (Figure 4B). With the growth of *Fusobacterium nucleatum*, the pH value of the bacterial suspension slowly increased but still appeared alkaline. In contrast, the pH value in the *Streptococcus mutans* suspension decreased to nearly 5.34, changing from alkaline to acidic.

## 4 DISCUSSION

Microorganisms and the human body are symbiotic because many microorganisms inhabit the human body. Once the ecological balance of the flora is destroyed, a series of diseases may occur. Accordingly, emerging researches have focused on the human microbial community, especially the intestinal flora. As the initial gateway of the digestive tract, the oral cavity directly contacts the outside environment and contains a diverse flora related to oral health.

In this study, a novel nucleic acid extraction method was first adopted to extract extremely small traces of microbiological DNA at the cervical dentin surface, and metagenomic sequencing was performed to solve the limit and make the extraction possible. The metagenomics method we utilized directly extracts total DNA from environmental samples and acquires information about the microbial community through sequencing, avoiding traditional research procedures of single clone isolation and culture. In fact, a large number of microorganisms in nature cannot be cultivated or obtained by artificial cultivation. As no culture is needed, the sequencing results can faithfully reflect the ecological flora. In addition, high‐throughput sequencing produces abundant data and low‐abundance microbial information in our study can also be obtained by increasing the data volume.

*Fusobacterium nucleatum*, an obligate anaerobic gram‐negative rod, has long been found to cause opportunistic infections in a wide spectrum of human diseases, such as oral infections^24^ and colorectal cancer.^25^ High content of *Fusobacterium* colonizes mainly the human oral cavity and has a symbiotic relationship with its hosts.^26^, ^27^, ^28^ As a conditional pathogen, *Fusobacterium nucleatum* not only contributes to forming healthy oral biofilms on the surface of normal teeth but also directly affects host immune responses to infection by other pathogens. It has a slender biological form has been demonstrated to extensively adhere to all oral microorganisms.^29^ In biofilms that form dental plaques, non‐motile *Fusobacterium nucleatum* can provide structural support as a bridge organism connecting primary colonizers. Every 10 *Streptococcus sanguis* could combine with one *Fusobacterium nucleatum* to form a highly ordered corncob structure when cocultured.^30^ Other researchers found it could synthesize and secrete a variety of lectins, which have strong coagulation and copolymerization effects with other bacteria, playing indispensable roles in plaque biofilm formation, bacterial colonization and mixed infection.^31^ For *Streptococcus mutans* and *Fusobacterium nucleatum*, RadD‐SpaP has been described as the interacting adhesin pair for binding.^32^ SpaP is the major *Streptococcus mutans* adhesin specific for binding to *Fusobacterium*, while RadD is a known *Fusobacterium nucleatum* adhesin mediating interaction with numerous gram‐positive species.^33^

When *Fusobacterium nucleatum* and *Streptococcus mutans* were mixed, the acid generated from *Streptococcus mutans* was speculated to be neutralized by *Fusobacterium nucleatum*, which resulted in neutral pH. It is well known that the hard tissue of teeth is damaged by *Streptococcus mutans* via its destructive acid products. After culture after *Fusobacterium nucleatum*, the destruction of *Streptococcus mutans* was inhibited, suggesting that *Fusobacterium nucleatum* might play a protective role by neutralizing the destructive acid.

To conclude, the wedges were confirmed to have a certain correlation with the distribution of microorganisms in our research. Although there was no significant difference between the distribution and diversity of the microbial population at the defect site and that on normal teeth, statistical analysis of each bacteria using a paired *t* test revealed significant differences in *Actinomycetales*, *Clostridiales* and *Fusobacteriales*, among which the difference in *Fusobacteriales* was the most significant. The most common oral flora constituent *Fusobacterium nucleatum*, a *Fusobacteriales* species, was selected to further explore its interaction with cervical defects, and a hypothesis suggesting protection of the tooth neck by *Fusobacterium nucleatum* was proposed. It is speculated that *Fusobacterium nucleatum* may protect the tooth neck by strong adhesion with other destructive bacteria, as well as neutralization of the destructive acid by increasing the pH of the bacterial suspension. The above connection and studies indicated the importance of *Fusobacterium nucleatum* research, which could provide a new perspective and evidence for the relation between microorganisms and NCCLs, and guide clinical treatment as well as preventive dentistry.

## CONFLICT OF INTEREST

The authors declare no competing interest.

## AUTHOR CONTRIBUTION

**Xiaoyu Huang:** Conceptualization (equal); Data curation (equal); Formal analysis (equal); Methodology (equal); Validation (equal); Writing‐original draft (equal). **Lin She:** Investigation (equal); Methodology (equal); Software (equal). **Huanhuan Liu:** Investigation (equal); Methodology (equal); Software (equal). **Pingping Liu:** Data curation (equal); Formal analysis (equal); Investigation (equal). **Jue Chen:** Data curation (equal); Formal analysis (equal); Investigation (equal). **Yingcong Chen:** Data curation (equal); Software (equal). **Wenjie Zhou:** Data curation (equal); Software (equal). **Jun Lin:** Conceptualization (equal); Funding acquisition (equal); Project administration (equal); Writing‐review & editing (equal). **Youguang Lu:** Conceptualization (equal); Funding acquisition (equal); Project administration (equal); Writing‐review & editing (equal).

## ETHICAL APPROVAL

This study was approved by the Institutional Review Board of School and Hospital of Stomatology, Fujian Medical University, and written informed consent was obtained from all subjects before interviews.

## Data Availability

The sequencing raw data reported in this article have been uploaded to the NCBI‐SRA Database under the accession number of PRJNA589106.
